# Comparative Evaluation of Bandage Contact Lenses and Eye Patching after Bilateral Cataract Surgery

**DOI:** 10.1155/2021/2873543

**Published:** 2021-08-13

**Authors:** Dalan Jing, Aihua Deng, Hongmei Wang, Yilin Chou, Xiaodan Jiang, Zhenxiang Chen, Xuemin Li, Tingyi Wen

**Affiliations:** ^1^Department of Ophthalmology, Peking University Third Hospital, Beijing, China; ^2^Beijing Key Laboratory of Restoration of Damaged Ocular Nerve, Peking University Third Hospital, Beijing, China; ^3^CAS Key Laboratory of Pathogenic Microbiology and Immunology, Institute of Microbiology, Chinese Academy of Sciences, Beijing, China

## Abstract

**Purpose:**

To comparatively evaluate the safety and satisfaction of bandage contact lens (BCL) and eye patching in patients after cataract surgery.

**Methods:**

Sixteen (32 eyes) patients who planned to undergo bilateral cataract surgery were recruited. The two eyes of each patient were randomly divided into 2 groups. Group A and Group B were instructed to wear BCLs immediately at the end of the surgery until one week and eye patch immediately after surgery until one day, respectively. Visual analog scales of ten specific symptoms, Visual Function Index (VF-14) questionnaire, and best-corrected visual acuity (BCVA) were conducted on the first day before the surgery and Day 1 and Day 7 after surgery. Oculus keratography was conducted on the first day before surgery and on Day 7. Patient satisfaction was determined on Day 1. Moreover, bacterial species in the conjunctival sac, meibomian gland secretions, and BCLs were subsequently identified using 16S rRNA gene sequencing.

**Results:**

The patient satisfaction scores of Group A were higher than Group B. Group A were more motivated to choose the same treatment and were more likely to recommend BCLs to others. No statistically significant differences were found in bacterial culture positivity between the groups. The differences in ocular signs and symptoms between the two groups were not statistically significant. There were no significant differences in the BCVA and VF-14 between the groups at any time point.

**Conclusions:**

BCLs could be safely and effectively used in patients after cataract surgery.

## 1. Introduction

Phacoemulsification shows significantly improved visual acuity for patients with cataract. Today the routine postoperative management is wearing an eye patch overnight, which has been considered as an efficient method for preventing infection. Unfortunately, it is associated with inconvenience and even injury by monocular vision and stereopsis defects [1]. In addition, eye patching after cataract surgery did not reduce postoperative discomfort and inflammation compared to “instant vision” without patching. [2] A trend towards no patching cataract surgery was observed especially with modern surgical technologic improvements. The “instant vision” obtained for postoperative patients is of particular importance especially for the fellow eye of blind or poor visual acuity.

Bandage contact lenses (BCLs) are widely used for corneal diseases (corneal lesions, corneal refractive surgery, and corneal transplantation) to reduce pain, provide mechanical and structural protection, promote epithelial healing, and accelerate visual rehabilitation [3–6]. BCLs may prolong drug release and retention towards the post-lens tear fluid, which could be useful in the treatment of ocular surface disease. [7].

However, contact lenses provide a suitable substratum for bacterial adherence which might lead to contact-lens-related infections; [8] the lens also limits the tear fluid exchange that reduces antimicrobial tear fluid perfusion [9]. The clinical safety of BCLs remains controversial [10–12], and additional studies are required to better evaluate the BCLs. Hence, a subsequent paired comparison was performed to evaluate the safety and satisfaction of BCLs.

## 2. Materials and Method

### 2.1. Participants

This study was conducted according to the principles of the Declaration of Helsinki and was approved by the Human Research and Ethics Committee of Peking University Third Hospital (16-08-QX-YK). Written informed consent in Chinese was obtained from each participant before recruitment.

In this prospective clinical study, 16 (32 eyes) patients with bilateral age-related cataract who were willing to undergo outpatient phacoemulsification and intraocular lens implantation surgery within one week were recruited. Besides, each patient experienced two approaches in order to facilitate the clinical comparison between BCLs and eye patching. Patients were not influenced in any way to consider one method may be superior to the other to avoid any possibly psychological biasing effects. Patients with contact lens wear within 1-month, recent eye surgery, nasolacrimal duct obstruction, corneal diseases, eye trauma, eye infection, glaucoma, and ocular fundus diseases were excluded from the study population.

### 2.2. Examinations Procedure

The clinical assessments of the enrolled participants were conducted in the following order at the clinical first visit: collection of demographic information, best-corrected visual acuity (BCVA), Visual Function Index (VF-14) questionnaire, [13] visual analog scales of 10 specific symptoms which were used to assess the patient's subjective symptoms (dryness, foreign body sensation, ache, burning, tearing, asthenopia, blur, itching, secretions, and photophobia) [14], painful hours, sleep quality [15], and slit-lamp examination (conjunctival injection, subconjunctival hemorrhage, corneal edema, keratic precipitate, and anterior chamber flare) which were used as postoperative inflammation indicators. The Keratograph 5M (Oculus Optikgeräte GmbH, Wetzlar, Germany) was used to measure the noninvasive tear break-up time (TBUT) and tear meniscus height (TMH) on the first day before the surgery and on Day 7. It is important to note that the Keratograph was conducted at 30 min following BCL removal to reduce the influence of ocular procedures at Day 7. Visual analog scales of 10 specific symptoms on the first day before the surgery, Day 1, and Day 7 were collected. Satisfaction scores (Table 1) were assessed on Day 7. Slit-lamp microscope examination was carried out at each visit by the same doctor, and the signs including subconjunctival hemorrhage, conjunctival congestion, corneal swelling, keratic precipitates, and anterior chamber flare were evaluated using a dichotomous scale and recorded as absent or present.

### 2.3. Surgical Technique

All patients were given levofloxacin eye drops (Santen, Japan) four times a day from three days before surgery. Different eyes in the same patient were divided randomly into two groups. Povidone-iodine (PI) 5% solution was instilled before and after placement of the lid speculum. All underwent standard phacoemulsification through a 3.2 mm clear corneal incision and intraocular lens implantation by the same surgeon (LXM). After surgery, patients were instructed to wear BCLs made of balafilcon A (water content 36%; extended wear; Pure Vision; Bausch&Lomb Inc, Rochester, NY) for a week, did not receive any antibiotic ointments when the surgery was over (Day 0), and were prescribed antibiotic eyedrops for a month from Day 0. Group B received antibiotic ointment at Day 0, then were instructed to wear the eye patch, and were given antibiotic eyedrops for a month from Day 1.

### 2.4. Samples Collection

Preoperative samples were collected in a sterile treatment room by the sample ophthalmologist (JDL) who wore sterile gloves and mask. Sterile swabs were used to collect bacterial samples from the lower conjunctival sac (CS) and meibomian gland (MG) secretions on the first day before surgery. The bacterial samples from the lower CS, MG secretions, and corneal incision site were acquired immediately at the end of surgery. The lower CS, MG secretions samples were collected at Day 1 and Day 7. Moreover, the samples of the corneal incision site and the disposed BCLs were obtained at Day 7. The BCLs were removed at the slit lamp using sterile forceps without topical anesthesia. All the samples were placed in the sterile Eppendorf tubes (Axygen, USA) and stored at 4°C.

### 2.5. Bacterial Culture and Phylogenetic Analysis of 16S rRNA Sequence

Microbial culture and identification were conducted based on previous studies [16]. The samples were inoculated in blood-agar media within 24 hours of collection. After a 72-hour incubation at 37°C, colonies with different phenotypes were selected for further analysis. The 16S rRNA gene was amplified by using colony PCR using primers 27F and 1492R [17, 18]. PCR products were sequenced by Tianyi Huiyuan Biotechnology Company (Beijing, China). Correct species identification was obtained by comparing the 16S rRNA gene sequences and the sequence similarities were analyzed using the Basic Local Alignment Search Tool (BLAST, https://blast.ncbi.nlm.nih.gov/Blast.cgi). For phylogenetic analysis, the 16S rRNA sequences of all strains were aligned using the closely related species as references using CLUSTAL_W. [19] The maximum-likelihood [20] was selected to construct the phylogenetic tree using MEGA version 7.0. [21].

### 2.6. Statistical Analysis

All the analyses were performed using the SPSS version 23.0 software. The Kolmogorov-Smirnov test was applied to determine the data normality. The descriptive data are presented as mean and standard deviations (SD). The independent *t* test was adopted to compare the differences between the two groups for continuous variables and the Wilcoxon nonparametric test was applied for ordinal variables. Statistical significance was set at *p* < 0.05.

## 3. Results

### 3.1. Demographics

A total of 16 patients were included in the baseline of the study. The average age of patients was 74.8 ± 6.7 y (range, 64–86 y; 8 women).

### 3.2. Visual Function

There were no differences in the BCVA and VF-14 between the two groups at baseline or at follow-up visits (*p* > 0.05). However, the BCVA and VF-14 of all the patients had improved compared to the levels prior to surgery.

### 3.3. Patient Subjective Symptom Evaluation

The differences in the patient subjective symptom scores (mean ± SD) between the two groups were not significant at baseline. There were no significant differences in patient subjective symptom scores between the two groups on the first postoperative day. No differences were observed in painful time and sleep quality. Moreover, subjective symptom scores showed no significant differences at the 1-week follow-up. After cataract surgery, patient subjective symptom scores decreased in both groups at Day 1 and Day 7; however, the differences were not significant except for asthenopia and blurred vision (Table 2).

### 3.4. Patient Objective Signs Evaluation

The differences in TBUT (mean ± SD) and TMH between the two groups were not significant at baseline (*p*=0.710, *p*=0.240). For both TBUT and TMH, there were no significant differences between Group A and Group B at the 1-week follow-up (*p*=0.706, *p*=0.482). Conjunctival injection, subconjunctival hemorrhage, keratic precipitates, and anterior chamber flare showed no statistically significant difference between the two groups at the 1-day and 1-week visit (Table 3). Besides, no serious complications were found in all patients such as acute conjunctivitis, keratitis, endophthalmitis, anaphylaxis, and corneal epithelial injury.

### 3.5. Phylogenetic Analysis of Isolated Bacteria

**Figure 1:**
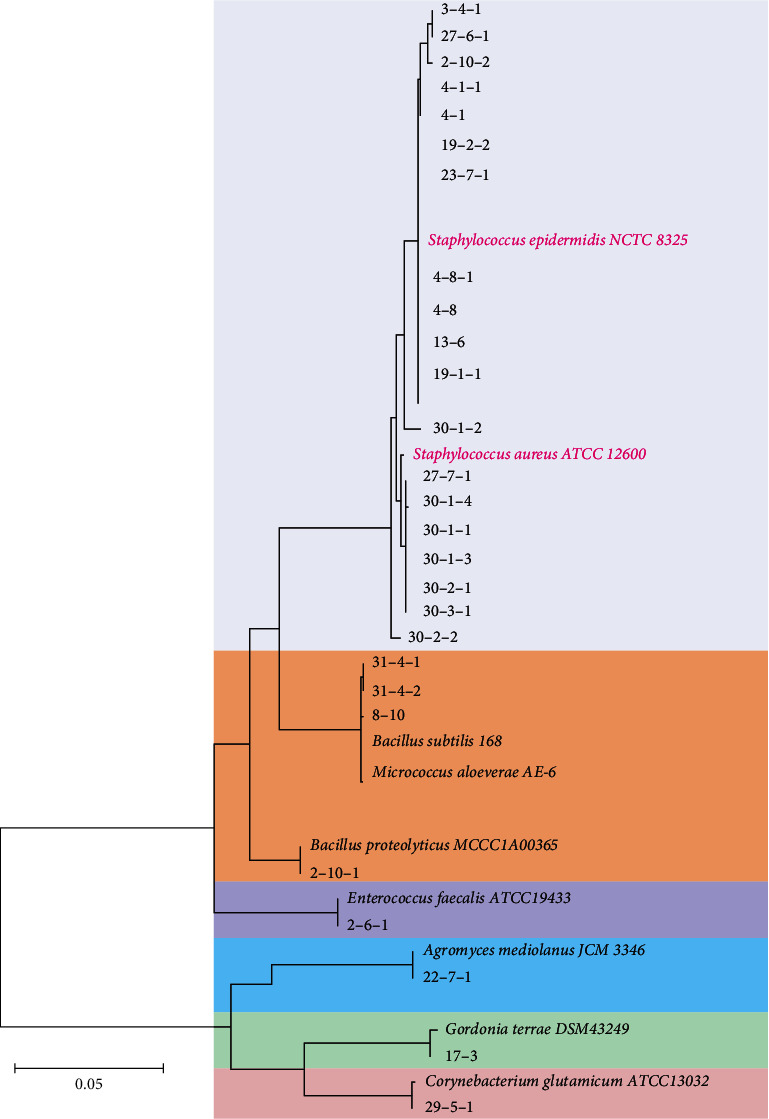
Phylogenetic tree based on 16S rRNA gene sequences. Maximum-likelihood phylogenetic tree is constructed based on the bacterial 16S rRNA gene sequences. Numbers at the nodes are ML bootstrap proportions. Bar represents 0.05 substitutions per nucleotide position.

Phylogenetic analysis revealed that all isolated bacteria were divided into genus *Staphylococcus*, *Bacillus*, *Micrococcus*, *Agromyces*, *Gordonia*, *Enterobacter*, and *Corynebacterium* (Figure 1). Most of bacteria with high similarities belong to the Gram-positive *Staphylococcus*, including *S. epidermidis*, *S. aureus*, *S. hominis*, and *S. lugdunensis*. Some bacteria belong to the Gram-positive *Bacillus*, including *B. amyloliquefaciens*, *B. proteolyticus*, *B. tequilensis*, and *B. velezensis*. A few bacteria were belonging to the *A. mediolanus* (*G*^+^), *M. aloeverae* (*G*^+^), *G. hongkongensis* (*G*^+^), *C. glutamicum* (*G*^+^), and *E. faecalis* (*G*^−^). The phylogenetic analysis indicated that most isolated strains from the patients' eyes were within the Gram-positive *Staphylococcus* and *Bacillus*.

### 3.6. Safety Evaluation Based on Bacterial Culture and Identification

The positive rate of bacterial cultures in different swabs is shown in Table 4. Group A did not differ significantly from Group B in culture positivity. The microbiomes isolated from the samples are shown in Table 5. In both the CS and MG secretions, most of the isolated bacteria were Gram-positive, including *Staphylococcus epidermidis and Staphylococcus aureus.* Two cases that were culture-positive were undetectable by the 16s rRNA Gene Sequencing, which were termed “Fail,” and warranted further characterization.

### 3.7. Patient Satisfaction

The patient satisfaction score of Group A was higher than Group B but was not statistically different (8.38 ± 1.59 vs. 7.63 ± 1.75, *p*=0.213); 75% of the patients preferred wearing the BCLs, while only 25% of the patients showed equivalent preference for eye patching. Similarly, they were willing to recommend the same approaches to their friends.

## 4. Discussion

Currently, eye patching in the postoperative period of cataract remains routine regimen. Until recently, tight eye patching following placement of ointment could increase the risk of toxic anterior segment syndrome [22]. In addition, eye patching following routine cataract surgery is associated with an increase in corneal edema and slower visual recovery on the first postoperative day [23]. More ophthalmologists have recognized that patching may be not the best choice for patients [2]. It has been shown that no differences in safety in the postoperative management of cataract are observed regardless of patching; thus, further efforts are to be directed towards enhancing patient comfort and acceptance [24]. The silicone hydrogel BCL has higher oxygen permeability and water absorbability; thus, it has been used extensively in ocular surface diseases [12, 25]. Motivated patients with bilateral age-related cataract were recruited who were willing to undergo cataract surgery at different days, in order to ensure accuracy regarding the satisfaction and safety of BCLs, which are used after cataract surgery, and pairwise comparison was conducted to reduce bias.

Visual analog scale rather than the ocular surface disease index (OSDI) questionnaire was used to measure the syndromes since OSDI cannot distinguish the concerned eye and cannot accurately assess syndromes. There has been much debate about whether wearing BCLs can reduce postsurgery discomfort such as foreign body sensation [1, 10, 26]. We noticed that differences between these groups regarding postsurgery discomfort did not change significantly. Furthermore, the painful time and sleep time showed no distinction. All the subjective symptom scores decreased from baseline, Day 1, Day 7 in both the groups postoperatively; however, only asthenopia and blurred vision showed significant improvement. Discomfort following cataract surgery and particularly the eye pain and foreign body sensation were due to contact between the eyelid and the corneal incision or epithelial damage. Eye patching could restrict the motion of eyelid by increasing the willing to close the eye and therefore enhance epithelial repair and relieve pain or foreign body sensation. BCLs cover the corneal surface thus reducing exposure between the eyelid and cornea and protect the cornea from exposure or from the irritation caused by rubbing the eye, thereby facilitating corneal epithelium healing [27]. Silicone hydrogel materials with high oxygen transmissibility, specifically designed for continuous wear, could secure enhanced wound healing and epithelial cell reproduction [28]. All the abovementioned factors reduced postoperative pain and foreign body sensation.

Neither group showed any differences in the postoperative inflammation indicators at Day 1 and Day 7, which were consistent with previous studies [1, 10]. No cases of severe corneal injury or postoperative inflammation were identified. Hence, BCL and patching both may be considered valuable methods in the postoperative regime, offering a comparable clinical alternative. The results suggested that no significant differences of TBUT and TMH were observed between these groups at Day 1 and Day7, which varied from previous reports. Other studies showed that BCLs played a positive role in stabilizing TBUT and improving TMH [1, 10, 26]. This could be attributed to the differences of measurements. Keratograph was used, which permits an automated, hypersensitivity, and examiner independent technique for measuring TBUT. TBUT as measured using the Keratograph was consistently lesser than the subjective observer recordings since it can record the first incident of break-up anywhere in the tear film [29]. Silicone hydrogel contact lenses are known to aid in stabilizing the tear film, permit corneal healing, and restore normal cell turnover, all of which are critical to the treatment of ocular surface diseases [30]. Similar findings were reflected in our study. While differences in the TBUT and TMH between eye patching and BCL were not observed, it is interesting to speculate that short-term wearing of BCLs may not be sufficient to produce changes. The ability to stabilize the tear film could be transient; hence, these changes could not be captured since the TBUT were measured following 30 min of extracting the BCLs. However, others were subjectively measured immediately and could not avoid the irritant effects of ocular procedures.

Extended and overnight soft contact lens wear has been identified as a risk factor for corneal infection. [31] Endophthalmitis is the most severe complication following cataract surgery. Assessing the presence of bacteria is crucial for using BCLs in postoperative cataract surgery. A previous study has evaluated the safety of overnight BCLs through bacterial culture and biochemical identification, [10] although conclusions were limited by short-term wearing, which could not indicate the safety and efficacy for long-term use. Bacterial incubates were obtained from different parts at selected time points and continuous samples (one-week period) using BCLs were obtained. The bacterial culture positivity showed no statistically significant differences between these two groups. The organisms most frequently cultured were *Staphylococcus epidermidis and Staphylococcus aureus.* The present study indicates that selecting BCLs following cataract surgery is safer compared to eye patching. Consistent with previous studies [32], preoperative prophylactic antibiotics are an effective treatment despite revealing the presence of bacteria in a small number of samples. It was of interest to note that some eyes with preoperative negative cultures were immediately positive following surgery no matter in CS, MG, or corneal major incision. This could be since MG secretions continue during the operations, especially after lid speculum placement [33]. Moreover, Group B showed more positive results in the cultures of postoperative Day 1. Povidone-iodine is reportedly effective in reducing conjunctival bacterial flora [34]. It has been shown that a small amount of PI remains in the conjunctival sac at the time of operation, which could prevent bacterial contamination [33]. A recent study reported that the 0.66% PI eye drops used for three days prior to cataract surgery were effective in reducing the conjunctival bacterial load [35]. Moreover, Oliverio et al. [36] hypothesized that PI acting on ocular surface microbiota may rebalance the anomalous bacterial overgrowth typical of DED, resulting in a reduction of the inflammatory stimulus on the ocular surface epithelial cells, thus relieving symptoms of dry eye.

The polymer structure of the soft BCLs could act as a reservoir, which could prolong the duration of antibiotic eyedrops or PI. Although PI was considered to possess corneal toxicity, which depends on the concentration, diluted PI had a higher bactericidal efficiency owing to greater availability of diatomic free iodine in dilute solution, the bactericidal component of PI [34]. And PI seems to contain factors that may favor the ocular surface protection such as glycerol and vitamin *E* d-alpha-Tocopherol Polyethylene Glycol (vit ETPGS) [36]. Corneal toxicity and DED protection in low-concentration PI retention of BCLs warrant further study.

Recent researches [3, 37] have shown that the silicon material also exhibits a good capacity for water absorption and BCLs application to the eye, causing the stagnation of the liquid beneath the BCLs. Therefore, the silicon could help lock in water mimicking the lipid layer, thereby reducing tear evaporation, and locking drugs possible. Hence, patients should receive antibiotics in the immediate postsurgical period. BCLs provide a convenient method for this intervention. It is particularly interesting that *Enterococcus faecalis* was seen in patient 2 of Group B. *Enterococcus* is Gram-positive cocci usually found in the normal human gastrointestinal tract and is an uncommon cause of endophthalmitis. [38] However, it accounted for 2.2% of the culture-proven acute-on-set postoperative endophthalmitis following cataract surgery or secondary intraocular lens implantation in the endophthalmitis in the endophthalmitis vitrectomy study [39]. Despite adequate interventions, the outcome of *Enterococcus* endophthalmitis was poor vision [40]. Treatment is complicated since it is highly resistant [41]. BCLs used in postoperative cataract did not show bacterial growth and there was no evidence of infection, which might be attributed to the antibiotic prophylaxis, high oxygen permeability of the BCLs, and autoimmunity to bacteria.

For the BCVA and VF-14 score, the difference was not statistically significant between the groups. Therefore, BCLs may not disrupt visual function. Regarding the satisfaction with the two regimes, subjective feeling was used to reflect the patients' satisfaction in previous studies. A more precise and quantitative means of satisfaction should be designed to indicate patient satisfaction. BCL group had a higher patient satisfaction score than the eye patching group, although the differences did not achieve statistical significance. Most patients (85%) showed more preference for BCLs, and the major reason was instant vision, which is advantageous for normal life and reducing accident. Clearly, instant vision provides better orientation and improved vision immediately following surgery, which was obtained from the BCLs. These findings led to more patients opting for ambulatory surgery with BCLs that reduced hospitalized anxiety. Twenty-five percent of the patients chose eye patching since they believed patching may provide more protection and more rest for the eyes. All these data supported the hypothesis that BCLs could be a good alternative to eye patching. The main contributions of this paper are summarized as follows: We recruited motivated patients with bilateral age-related cataract that can more accurately reflect the truth about the satisfaction and safety of BCLs. We developed bacterial cultures obtained from different sites and at various time points perioperatively that can reveal the alteration in the ocular flora.

The present study has several limitations. First, the sample size was small; further studies with larger number of patients are needed. Although the study sample size also was relatively small, we found the bacterial culture positivity of the two groups showing no difference resembled the former result. Second, only aerobic bacteria isolated from the ocular surface were explored, and further studies should also include anaerobic bacteria. Third, a further analysis is needed for bacterial identification, such as correlation analysis.

## 5. Conclusion

In conclusion, we consider that BCLs could offer a better alternative to eye patching in the postoperative cataract regime with higher patient satisfaction and equal safety.

## Figures and Tables

**Table 1:** Satisfaction questionnaire of postoperative cataract regime.

Item	Score (0–10)/choice
Patient satisfaction score of eye patching	
Patient satisfaction score of bandage contact lenses	
The preference for which regime of second eye	
The willing to recommend which regime to your friends	

**Table 2:** Patient subjective symptom scores of patients.

		Baseline			Day 1 vs baseline			Day 7 vs baseline		
		Group *A*	Group *B*	*P*	Group *A*	Group *B*	*P*	Group *A*	Group *B*	*P*
Symptom	Dryness	2.94 ± 2.91	2.75 ± 2.91	NS	1.25 ± 1.95	1.56 ± 2.37	NS	2.00 ± 2.90	0.75 ± 1.73	NS 0.026
	Foreign body sensation	2.31 ± 3.40	2.25 ± 3.47	NS	1.38 ± 2.36	2.81 ± 3.60	NS	1.94 ± 2.54	1.00 ± 1.71	NS
	Burning	0.25 ± 0.77	0.25 ± 0.77	NS	0	0	NS	0	0	NS
	Tearing	1.81 ± 2.64	1.81 ± 2.64	NS	1.63 ± 2.92	1.25 ± 1.98	NS	0.69 ± 1.82	0.69 ± 1.82	NS
	Asthenopia	3.56 ± 2.71	3.56 ± 2.71	NS	1.44 ± 2.66	0.53 ± 1.45	0.033 0.001	2.06 ± 2.17	2.00 ± 2.07	NS
	Blurred vision	6.56 ± 2.31	5.94 ± 2.41	NS	4.88 ± 3.59	3.39 ± 2.66	NS 0.012	2.69 ± 2.85	2.63 ± 2.78	0.000 0.001
	Itching	2.38 ± 3.16	2.19 ± 3.21	NS	0.56 ± 1.75	0.67 ± 1.40	NS	1.06 ± 1.95	1.06 ± 1.95	NS
	Secretions	2.63 ± 2.75	2.00 ± 2.48	NS	1.38 ± 2.58	0.5 ± 1.10	NS 0.038	1.13 ± 1.41	1.25 ± 1.65	NS
	Photophobia	2.38 ± 2.99	2.31 ± 2.98	NS	1.53 ± 2.17	0.53 ± 1.13	NS 0.038	1.19 ± 2.04	1.27 ± 2.09	NS
	Ache	2.06 ± 2.99	2.19 ± 3.00	NS	1.00 ± 1.90	1.36 ± 2.58	NS	1.13 ± 1.78	0.88 ± 1.75	NS

NS: no significant differences.

**Table 3:** Outcomes of postoperative inflammation indicators.

Time points	Postoperative Day 1			Postoperative Day 7		
	Group *A n* (%)	Group *B n* (%)	*P*	Group *A n* (%)	Group *B n* (%)	*P*
Subconjunctival hemorrhage	2 (12.5)	4 (25)	0.37	1 (6.25)	1 (6.25)	NS
Conjunctival injection	15 (93.75)	16 (100)	0.16	0 (0)	1 (6.25)	NS
Corneal edema	11 (68.75)	11 (68.75)	1	0 (0)	0 (0)	NS
Keratic precipitate	16 (100)	16 (100)	1	0 (0)	0 (0)	NS
Anterior chamber flare	16 (100)	16 (100)	1	8(50)	10 (62.5)	NS

NS: no significant differences.

**Table 4:** The incidence of positive bacterial cultures and species distribution.

Sample' sources	Group *A* positive culture rate (patient)	Group *B* positive culture rate (patient)	*P*
Pre-op CS	6.25% (4)	12.5% (19,30)	NS
Pre-op MG	6.25% (4)	12.5% (19,30)	NS
Post-op Day 0 CS	0	12.5% (17,30)	NS
Post-op Day 0 MG	12.5% (3,31)	0	NS
Post-op Day 0 corneal incision	0	6.25% (29)	NS
Post-op Day 1 CS	6.25% (13)	12.5% (2,27)	NS
Post-op Day 1 MG	0	18.75% (22,23,27)	NS
Post-op Day 7 CS	6.25% (4)	0	NS
Post-op Day 7 MG	0	0	NS
Post-op Day 7 corneal incision	0	12.5% (2,8)	NS
Post-op Day 7 BCL	0	—	—

CS: conjunctival sac. MG: meibomian gland. BCL: bandage contact lens. Pre-op: before surgery. Post-op: after surgery. NS: no significant differences.

**Table 5:** Bacteria isolated in samples among Group A and Group B.

Isolated bacteria in Groups A and B		Pre-op CS	Pre-op MG	Post-op Day 0 CS	Post-op Day 0 MG	Post-op Day 0 corneal incision	Post-op Day 1 CS	Post-op Day 1 MG	Post-op Day 7 CS	Post-op Day 7 MG	Post-op Day 7 corneal incision	Post-op Day 7 BCL
*A*	Patient 3	—	—	—	*S. epidermidis*	—	—	—	—	—	—	—
	Patient 4	*S. epidermidis*	Unknown	—	—	—	—	—	*S. epidermidis*	—	—	—
	Patient 13	—	—	—	—	—	*S. epidermidis*	—	—	—	—	—
	Patient 31	—	—	—	*B. amyloliquefaciens B. velezensis*	—	—	—	—	—	—	—

*B*	Patient 2	—	—	—	—	—	*E. faecalis*	—	—	—	*B. proteolyticus S. epidermidis*	—
	Patient 8	—	—	—	—	—	—	—	—	—	*B. tequiensis*	—
	Patient 17	—	—	*Gordonia hongkongensis*	—	—	—	—	—	—	—	—
	Patient 19	*S. epidermidis*	*S. epidermidis*	—	—	—	—	—	—	—	—	—
	Patient 22	—	—	—	—	—	—	*Agromyces mediolanus*	—	—	—	—
	Patient 23	—	—	—	—	—	—	*S. epidermidis*	—	—	—	—
	Patient 27	—	—	—	—	—	*S. epidermidis*	*S. aureus*	—	—	—	—
	Patient 29	—	—	—	—	*Corynebacterium glutamicum*	—	—	—	—	—	—
	Patient 30	*S. aureus*	*S. aureus S. haemolyticus*	*S. aureus*	—	—	—	—	—	—	—	—

CS: conjunctival sac. MG: meibomian gland. BCL: bandage contact lens. Pre-op: before surgery. Post-op: after surgery.

## Data Availability

The data used to support the findings of this study are available from the first author upon request.
